# Association of Kiss1 and GPR54 Gene Polymorphisms with Polycystic Ovary Syndrome among Sri Lankan Women

**DOI:** 10.1155/2019/6235680

**Published:** 2019-03-12

**Authors:** Umayal Branavan, Kajan Muneeswaran, W. S. S. Wijesundera, Anoma Senanayake, N. Vishvanath Chandrasekharan, Chandrika N. Wijeyaratne

**Affiliations:** ^1^Department of Obstetrics and Gynecology, Faculty of Medicine, University of Colombo, Colombo, Sri Lanka; ^2^Department of Chemistry, Faculty of Science, University of Colombo, Colombo, Sri Lanka; ^3^Department of Biochemistry and Molecular Biology, Faculty of Medicine, University of Colombo, Colombo, Sri Lanka; ^4^De Soysa Maternity Hospital, Colombo, Sri Lanka

## Abstract

Polycystic ovary syndrome (PCOS) is the commonest endocrine disorder affecting women of reproductive age. Its aetiology, though yet unclear, is presumed to have an oligogenic basis interacting with environmental factors. Kisspeptins are peptide products of Kiss1 gene that control the hypothalamic pituitary (HPG) axis by acting via G protein-coupled receptor known as GPR54. There is paucity of data on the role of Kiss1 and GPR54 gene in PCOS. We aimed to identify the polymorphisms in Kiss1 and GPR54 genes and explore their association with serum kisspeptin levels among Sri Lankan women with well-characterized PCOS. Consecutive women with PCOS manifesting from adolescence (n=55) and adult controls (n=110) were recruited. Serum kisspeptin and testosterone levels were determined by ELISA method. Whole gene sequencing was performed to identify the polymorphisms in Kiss1 and GPR54 genes. Serum kisspeptin and testosterone concentrations were significantly higher in women with PCOS than controls: kisspeptin 4.873nmol/L versus 4.127nmol/L; testosterone 4.713nmol/L versus 3.415 nmol/L, p<0.05. Sequencing the GPR54 gene revealed 5 single nucleotide polymorphisms (SNPs), rs10407968, rs1250729403, rs350131, chr19:918686, and chr19:918735, with two novel SNPs (chr19:918686 and chr19:918735), while sequencing the Kiss1 gene revealed 2 SNPs, rs5780218 and rs4889. All identified SNPs showed no significant difference in frequency between patients and controls. GPR54 gene rs350131 polymorphism (G/T) was detected more frequently in our study population. The heterozygous allele (AG) of GPR54 gene novel polymorphism chr19:918686 showed a marginal association with serum kisspeptin levels (p=0.053). Genetic variations in GPR54 and Kiss1 genes are unlikely to be associated with PCOS among Sri Lankan women manifesting from adolescence. Meanwhile the heterozygous allele of chr19:918686 is probably associated with serum kisspeptin concentrations, which suggests a potential role in the aetiology of PCOS.

## 1. Introduction

Polycystic ovary syndrome (PCOS) is a common endocrine disorder affecting women of reproductive age. Its community prevalence ranges between 2% and 15% in differing geographic locations and ethnic groups, particularly in its age specific prevalence [1]. The community prevalence of PCOS in young pre-marital Sri Lankan women is 6.3 % [2].

Female reproductive function depends on proper development and regulation of the hypothalamic pituitary gonadal (HPG) axis. Kisspeptins are peptide products of Kiss1 gene that participate in controlling the HPG axis. Kisspeptin acts via the G protein-coupled receptor known as GPR54 [3, 4]. The GPR54-Kiss1 pathway has an essential role in the initiation and maintenance of mammalian fertility [5]. Kiss1 gene was first identified by Lee et al. (1996) as a metastasis suppressing gene in melanoma cell line [6]. This discovery added a new dimension to understanding the physiology of HPG axis, reproduction and fertility [7, 8].

Kisspeptin signals GnRH neurons directly through its action on the kisspeptin receptor (GPR54) to release GnRH into the portal circulation, which in turn stimulates the secretion of LH and FSH from the gonadotrophs of the anterior pituitary [9]. GnRH secretion is deregulated in PCOS. Therefore, it can be postulated that altered patterns of kisspeptin inputs to GnRH neurons lead to dysregulated gonadotropin secretion in PCOS. Since kisspeptin is produced from Kiss1 gene and acts via GPR54 receptor, variation in Kiss1 and GPR54 genes may result in alteration in serum kisspeptin levels and thereby gonadotropin secretion. The hormonal derangement in PCOS is gonadotropin dependent, with LH excess driven ovarian hyperandrogenism being the hallmark [10].

The Kiss1 gene is localized to chromosome 1q32 and consists of three exons, of which only part of the second and third exons are finally translated into a precursor 145 amino acid peptide, which is cleaved into three forms of kisspeptins containing 54, 14, or 13 amino acids. The three peptides exhibit the same affinity for their single receptor (GPR54), since they share a common C-terminal decapeptide. GPR54 maps to chromosome 19p13.3 and includes five exons, encoding a 398 amino acid protein with seven hydrophobic trans-membrane domains [11].

The Kiss1/GPR54 system plays an important functional role in the process of puberty. PCOS, in its well characterized form, commonly manifests from puberty and is linked to excess adiposity and insulin resistance (IR), particularly among South Asians. The temporal relationship of PCOS to puberty and the essential role of kisspeptin in puberty makes it necessary to explore mutations and polymorphisms in the GPR54 and Kiss1 genes and their association with PCOS. Polymorphisms in Kiss1 gene in relation to PCOS have not been reported until recently in Saudi women with PCOS [12].

Given the complex relationship between Kiss1/kisspeptin/GPR54 pathway and HPG axis, we aimed to identify the polymorphisms in Kiss1 and GPR54 genes and investigate the association between Kiss1 and GPR54 gene polymorphisms and serum kisspeptin levels in normal Sri Lankan women and those manifesting well characterized PCOS from adolescence. To the best of our knowledge, this is the first study to determine any association between both Kiss1 and its receptor gene (GPR54) polymorphisms and PCOS among South Asians.

## 2. Materials and Methods

### 2.1. Recruitment of Subjects

This study was approved by the Ethics Review Committee, Faculty of Medicine, University of Colombo, Sri Lanka. Written informed consent was obtained from all participants. Consecutive patients with well characterized PCOS manifesting from adolescence were recruited from the Endocrine Clinic of the University Obstetrics and Gynecology Department. Diagnosis of PCOS was based on the Rotterdam criteria [13], with the diagnostic certification made by a single clinical lead.

Sample size was calculated for a case-control design using Schlesselman method [14], as in our previous reports on other genetic aspects of the same study sample [15, 16].

The number of consecutive subjects with confirmed PCOS in adolescence was 50, to which 10% was added to make allowance for noncompliance and dropouts. Therefore, the number of cases was 55. By selecting double the number of controls per case, the total number of controls was 110.

### 2.2. Inclusion Criteria

Inclusion criteria were women whose symptoms manifested from adolescent years (11-19 years WHO), with all 3 diagnostic criteria present from 16 to 19 years of age [17].

Anovular PCOS or Amenorrhoea/Oligomenorrhea: Anovular cycles are defined when the cycle length is more than 35 days, and the lack of demonstrable ovulation by mid cycle and luteal phase ultrasound scans, and mid-luteal serum progesterone [17]. Amenorrhoea is absence of menstrual periods for six months or more in a woman who has previously been menstruating. Oligomenorrhea is menstrual periods occurring at intervals of greater than 35 days, with only four to nine periods in a year.

Polycystic ovaries on ultrasound: it is defined by transvaginal or transabdominal ultrasound scan of ovaries, performed within the first 5 days from the onset of menstruation, and finding 24 or more follicles per ovary, measuring between 2 and 9 mm and/or an ovarian volume >10 cm3 [13].

Hyperandrogenism is defined by clinical evidence of hirsutism by modified Ferriman-Gallwey score (mFG) ≥8, serum testosterone (T) > 3.5nmol/L and/or free androgen index (FAI) >5 [17].

### 2.3. Exclusion Criteria

Exclusion criteria included inherited disorders of IR such as Rabson–Mendenhall syndrome, Cushing syndrome, hyperprolactinaemia, untreated primary hypothyroidism, congenital adrenal hyperplasia, or an androgen secreting ovarian/adrenal tumor, those taking corticosteroid, antiepileptic, or antipsychotic drugs, history of hormonal contraception within the previous 6 months, pregnancy, and the first postpartum year.

Control sample: concurrently asymptomatic, normo-androgenic, normal cycling since adolescence, nonmedicated, consenting, community-based women of reproductive age in whom PCOS was objectively excluded by clinical, biochemical, and ultrasound assessment were recruited as controls. The control subjects were recruited from a single work setting where health promotion programs were conducted from 2012 (3 years before the study). Working women of similar ethnic and social background as the affected subjects were invited to participate in the study.

### 2.4. Clinical Evaluation

Clinical evaluation was by a questionnaire-based interview regarding socio demographic factors, detailed menstrual and obstetric histories, infertility if relevant, the onset and degree of clinical symptoms of PCOS, drug history, family history of diabetes, and other cardiovascular risk factors. Detailed physical examination included measurement of standing height to the closest centimeter [18] and weight in kilograms to calculate the BMI, waist and hip circumference and waist-to-hip ratio (WHR), resting blood pressure [19], hirsutism (mFG score), frontal balding, distribution of acne and acanthosis nigricans [20].

Evaluation of the modified FG score was done by a single medically qualified clinical lead of the Department of Obstetrics and Gynecology, Faculty of Medicine, University of Colombo.

Ultrasound examinations were performed by a single trained medically qualified ultrasonographer under the supervision of the Radiology lead of the De Soysa Hospital for Women, Colombo. Polycystic ovaries (PCOs) ovarian volume 10 cm3 and/or ≥ 24 of 2- 9 mm follicles in a single ovary by ultrasonography performed within a week of the last menstrual period [21].

### 2.5. Biochemical and Endocrine Evaluation

Biochemical and endocrine evaluation involved determining the kisspeptin and testosterone levels in serum by enzyme linked immunoassay kit (ELISA kit). Two millilitres of venous blood was collected into plain sterile tubes from each subject and serum was extracted. Serum kisspeptin and testosterone levels were measured with ELISA kits (Phoenix Pharmaceuticals Inc., Belmond, CA and Teco Diagnostics, USA, respectively) as per manufactures' recommendation. Concentration of the samples was determined from the standard curve of the known concentrations. Fasting blood glucose level was measured in all subjects. Routine laboratory tests performed to diagnose/monitor PCOS (follicular phase FSH, LH, thyroid stimulating hormone, and fasting blood glucose/75 g oral glucose tolerance test) which were carried out at the quality controlled laboratory of the National Hospital Colombo were recorded for PCOS subjects.

### 2.6. The Kiss1 and GPR54 Genes Analysis

DNA was extracted from blood samples (2mL) using Genomic DNA extraction kit (Promega, USA), following the manufacturer's protocol, and the DNA samples were subsequently stored at −20°C. The entire gene of Kiss1 (6,151 bases) and GPR54 (3,729 bases) genes was custom-sequenced by using Next Generation Sequencing method (LGC group, Germany).

### 2.7. Statistical Analysis

The Kolmogorov-Smirnov test was used to test the normality of distribution. Values with a biological distribution are presented as mean ± standard error for mean. Comparison of means between those with PCOS and controls was performed with the independent sample t-test for normal values. Comparisons between three groups were performed with multivariate general linear model based one-way analysis of variance (ANOVA). Difference in the distributions between groups was tested with chi-squared tests. All analyses were performed by SPSS software (v.18.0 SPSS, Inc., Chicago, IL). The level of significance was set as 5%. Deviations from the Hardy–Weinberg equilibrium were tested by comparison of observed and expected genotype frequencies with *χ*2 test. Calculation of genotype and haplotype associations for all the SNPs was carried out using SNPSTATS program (http://bioinfo.iconcologia.net/index.php?module=Snpstats). Five inheritance models (codominant, dominant, recessive, overdominant, and additive) were applied for statistical analysis. The best inheritance model was assessed using the Akaike information criteria (AIC) and the Bayesian information criteria (BIC) with the model with the lowest values being the best fit.

## 3. Results

### 3.1. Clinical Characteristics of the Study Population

Demographic, clinical, and hormonal characteristics of women with PCOS and controls are summarized in Table 1. The demographic characteristics of cases and controls were comparable, except the mean ages of the two groups being close to statistical significance (p=0.06). This difference may have been due to chance and unlikely to have affected the risk assessment, since age is usually not a confounder of gene testing. Women with PCOS had significantly higher BMI and mFG score. Serum kisspeptin and testosterone concentrations were significantly higher in women with PCOS versus controls: kisspeptin 4.873 versus 4.127 nmol/L; testosterone 4.713 versus 3.415 nmol/L (p<0.05).

### 3.2. Polymorphisms Identified in GPR54 and Kiss1 Gene Analysis

Sequencing of GPR54 and Kiss1 genes reveled 5 and 2 SNPs, respectively (Table 2). Among the 5 polymorphisms of GPR54 gene detected, three have been previously reported while two are novel (chr19:918686, A/G and chr19:918735, A/G). The two novel polymorphisms are located in the intron region (intron 2) of the GPR54 gene. The previously reported 3 SNPs of the GPR54 gene are located in exon 1 (rs10407968), intron 2 (rs1250729403), and intron 4 (rs350131). Sequencing of Kiss1 gene revealed two SNPs, located in the untranslated variant 5 prime end (rs5780218) and exon 3 (rs4889).

### 3.3. Genotypes Frequencies between Cases and Controls

Genotype counts and frequencies in PCOS and control groups are shown in Table 3. The frequencies of the identified polymorphisms were not significantly different between cases and controls. In the GPR54 gene rs10407968 (A/G) polymorphism, the frequency of heterozygous allele (AG) was higher in cases than in controls while the homozygous (GG) allele was present only in controls (8%). Furthermore, homozygous mutant allele (GG) was not detected in cases or controls in GPR54 gene rs1250729403 polymorphism and in the two novel polymorphisms (chr19:918686 and chr19:918735). Moreover, GPR54 gene rs350131 polymorphism (G/T) was detected more frequently in our study population (minor allele frequency = 42.65%). In Kiss1 gene polymorphisms rs5780218 and rs4889, the homozygous genotypes (CT/CT and GG respectively) were found less than those of their wild type and heterozygous genotypes in cases and controls.

### 3.4. Hardy-Weinberg Equilibrium and Haplotype Association Analysis

The total of 5 SNPs of GPR54 and 2 SNPs of Kiss1 gene were in accordance with Hardy-Weinberg equilibrium (HWE) in control subjects (p>0.05). Association between the identified SNPs and PCOS risk was analyzed under five gene models (codominant, dominant, recessive, overdominant, and additive).  Table 4 shows that the GPR54 gene novel polymorphism Chr19: 918735 was associated with an increased PCOS risk: in the recessive model, genotype GG (OR=2.53, 95% CI: 0.97-6.57, p=0.043). Moreover, the five gene models also showed no association between genotypes of Kiss1 SNPs and PCOS risk (Table 5). In addition, there was no significant association of PCOS with the haplotypes of GPR54 (rs10407968/ rs1250729403/ rs350131/ Chr19:918686/ Chr19:918735) and Kiss1 (rs5780218/ rs4889) (Tables 6 and 7).

### 3.5. Genotypes and Serum Kisspeptin Concentration

**Figure 1:**
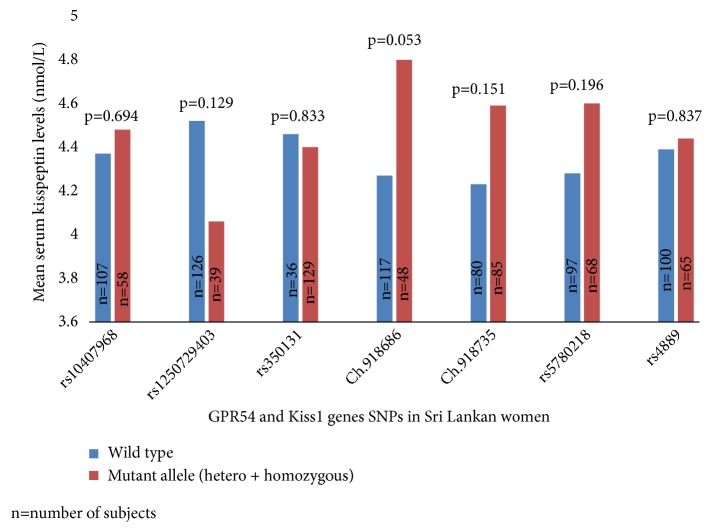
Association of serum kisspeptin levels with SNPs of GPR54 and Kiss1 genes.

The correlation of wild type and mutant alleles (heterozygous and homozygous) of each SNP with serum kisspeptin levels was analyzed. The identified polymorphisms, except chr19:918686 and serum kisspeptin level, did not differ significantly between PCOS subjects and controls (p>0.05). However, in the GPR54 chr19:918686 novel SNP, the heterozygous allele (AG) was marginally associated with serum kisspeptin levels when compared to the wild type allele (AA). Correlation between serum kisspeptin levels and wild type and mutant alleles (hetero + homozygous) of each SNP in cases and controls is summarized in Figure 1.

## 4. Discussion

The molecular mechanisms that trigger pubertal onset and modulate the inherent hormonal cascades are still being researched. Kisspeptin and its receptor GPR54 are the crucial regulators of the HPG axis in puberty. The GnRH neurons form the final pathway in regulating pituitary secretion of gonadotropins [22]. The activation of GnRH neurons is crucial for the onset of puberty [23]. GPR54 receptor is located on GnRH secreting neurons, suggesting that kisspeptin/GPR54 signaling within the GnRH neuronal network is important for pubertal activation and reproduction [24, 25]. Many studies have focused on Kiss1/GPR54 function, with only a few exploring any association between Kiss1 and GPR54 genes and PCOS. Several studies have reported mutations in GPR54 gene linked to idiopathic hypogonadotropic hypogonadism (IHH) [26–28] and central precocious puberty [29, 30], while mutations in Kiss1 gene have been linked to idiopathic CPP and normosmic IHH [31]. However, no definite causative mutation has been detected in GPR54 and Kiss1 genes in women with PCOS.

We found five SNPs in the GPR54 gene and two SNPs in the Kiss1 gene through sequencing. The frequency of each SNP when compared between PCOS and control groups showed no significant difference that was mirrored in the haplotype analysis. This suggests that the polymorphisms of GPR54 and Kiss1 genes do not individually confer a susceptibility to PCOS among a cohort of Sri Lankan women. The association of SNPs with PCOS gives confounding results in different studies. The lack of a demonstrable association between GPR54 and Kiss1 gene SNPs with PCOS can be due to multiple reasons.

The probable reasons for no genetic association being found include the following: (a) Need for an optimal sample size. Too small a sample can prevent the findings being scientifically extrapolated to the pathophysiology of PCOS, while too large a sample may falsely amplify the detection of a statistical difference that is clinically irrelevant. (b) Heterogeneous genetic etiology of PCOS, with the complex endocrine derangement caused by an interaction of susceptible and protective genomic variants of several genes being under the influence of environmental factors [32]. Hence, any variation in interlinking genetic factors might also affect the expression of GPR54 and Kiss1 gene, thereby showing no genetic association with PCOS. (c) Environmental interaction with the genetic predisposition may be responsible for the heterogeneity of the genetic etiology of PCOS. (d) Ethnicity, which has been demonstrated, leads to substantial ethnic/racial variation in the clinical expression of PCOS based on fetal programming, nutrition, and other aspects of differing locales [33]. A recent meta-analysis found that clinical associations may differ between ethnic populations due to different genetic background [34]. Therefore, we can assume that sample size, ethnicity, other genetic factors, and local environment might have contributed to the lack of association of GPR54 and Kiss1 gene SNPs with PCOS among Sri Lankan women.

Nevertheless, the GG genotype of GPR54 SNP (rs10407968) probably acts as a protective factor in PCOS (OR=0.893, 95% CI=0.829–0.962, p<0.05). Moreover, rs10407968 is a synonymous polymorphism (Gly→Gly) reported in girls with central precocious puberty (CPP) [35, 36]. However, there are no previous reports of an association of rs10407968 polymorphism with PCOS. Meanwhile, we did not find any association of PCOS with the previously reported intron variants (rs350131 and rs1250729403) and the two novel intron variants (Chr19:918686 and Chr19:9187350). Interestingly, GPR54 novel SNP chr19:918686 heterozygous allele (AG) showed marginal association with increased serum kisspeptin concentration (p=0.053) (homozygous allele GG was not present in chr19:918686 SNP).

Although introns are generally known as noncoding sections of genes, they can induce genetic and phenotypic variation by regulating or facilitating the transposition of exons during splicing [37]. There is increasing recognition of polymorphisms that modulate pre-mRNA processing, with at least 15% of point mutations related to human genetic disease caused by RNA splicing defects [38]. Mutations affecting pre-mRNA processing can be located in introns or exons, resulting in exon skipping or in the creation of new splice sites [39]. Most disease causing single nucleotide substitutions in donor or acceptor splice sites involve the +1/+2 or -1/-2 position, respectively [40]. However, pathological splicing alterations have also been observed to involve a variety of other positions, which can be close or fairly distant from these two pairs of canonical nucleotides. The functional reason why alterations in positions close to the splice sites may cause aberrant splicing is usually due to the disruption of interactions with U1 snRNP, U6 snRNP, and U2AF 65 or 35 in the splicing process [41]. The chr19:918686 SNP is located in the middle of the intron 2 and is less likely to have affected such an interaction. Hence, the mechanism behind chr19:918686 heterozygous allele and the observed marginal increase in serum kisspeptin level in our study population remains unclear.

We propose three possible mechanisms for the observed marginal increase in kisspeptin levels in the presence of chr19:918686 heterozygous allele (AG):

(1) If the chr19:918686 SNP is located within the regulatory sequences that can affect transcription of the remaining DNA, the resulting changes in the DNA sequence could affect the GPR54, such as a reduced number of receptors or a reduction in receptor affinity to kisspeptin. Such a functional impact can subsequently lead to increased circulating kisspeptin.

(2) The SNP (chr19:918686) causing a change in the RNA folding and thereby altering the secondary structure and splicing processes results in alteration of GPR54 receptor expression, which in turn increases circulating levels of kisspeptin.

(3) The increased kisspeptin levels with heterozygous allele (AG) of chr19:918686 are implicated in the pathogenesis of PCOS along with other genetic and environmental influences. Given the risk for developing PCOS being significantly influenced by environmental and other genetic factors and the human genetic code not changing significantly between two successive generations, the global secular trend in PCOS is due mostly to environmental and lifestyle changes. Genetic influence on the risk for developing PCOS is likely to be not only by direct alteration of Kiss1/Kissppetin/GPR54 interactions, but also by altering how any given individual interacts with environmental factors. Even within the same broad environment, individuals vary greatly in their adoption of unhealthy lifestyles and their willingness to change to a healthy lifestyle. A genetic influence on taste and food preferences, on willingness to change from unhealthy behaviors, on choosing options of either burning more calories or adopting a sedentary lifestyle is possible [42]. It is likely that genetic and environmental factors do play a role in determining serum kisspeptin levels in individuals with normal or variant allele.

We also found two SNPs in the Kiss1 gene in relation to PCOS– rs5780218 and rs4889, with rs5780218 resulting from the deletion of T base in the 5-prime untranslated region, a polymorphism not reported previously. The rs4889 polymorphism is found in the coding region and results in the substitution of the amino acid Proline by a basic amino acid Arginine. Such a change in the DNA sequence could alter the structure, function, and binding capacity of kisspeptin to its receptor GPR54. Previous studies in Chinese and Korean populations found no relationship between rs4889 and CPP [24, 43], which mirror our findings. Conversely, Fadwa et al. (2018) in a study of Saudi women conclude that a significant relationship exists between the rs4889 polymorphisms and PCOS [12]. Nevertheless, we found no association of the Kiss1 gene SNPs and serum kisspeptin levels in PCOS. We conclude that SNPs rs5780218 and rs4889 do not affect the synthesis of kisspeptin in PCOS.

## 5. Conclusion

The polymorphisms of GPR54 and Kiss1 genes have no significant association with PCOS in a cohort of Sri Lankan women, with well characterized phenotype manifesting from adolescence. Two novel polymorphisms, GPR54 SNPs chr19:918686 (A/G) and chr19:918735 (A/G), were detected with an association found between GPR54 SNP chr19:918686 heterozygous allele and a marginal increase in serum kisspeptin concentrations. The GPR54 gene rs350131 polymorphism (G/T) was detected more frequently in our study population. We propose that these novel findings may assist in providing a more scientific explanation for the differing expression of PCOS linked to genetic and environmental factors. Further study is recommended to explore possible mechanisms involving GPR54 and Kiss1 gene polymorphisms and their relationship with the expression of PCOS.

## Figures and Tables

**Table 1:** Demographic, clinical, and hormonal characteristic of the study population.

	PCOS	CONTROLS	p
	(n=55)	(n=110)	
Age (Years)	24.67 ± 0.883	33.80 ± 0.528	0.061
BMI(Kg/m^2^)	26.89 ± 0.716	25.25 ± 0.344	0.007
mFG score	8 ± 0.445	3 ± 0.222	0.006
WC:HC	0.839 ± 0.008	0.824 ± 0.004	0.114
FBG (mg/dL)	98.81 ± 2.08	108.69 ± 2.74	0.284
Kisspeptin (nmol/L)	4.873 ± 0.238	4.127 ± 0.132	0.033
Testosterone (nmol/L)	4.713 ± 0.458	3.415 ± 0.256	0.018
TSH (*μ*IU/mL)	1.96 ± 0.346		
FSH (mIU/mL)	5.5 ± 0.430		
LH (mIU/mL	7.34 ± 1.198		

BMI: body mass index; mFG: modified Ferriman-Gallway score; WC:HC: waist circumference: hip circumference; FBG: fasting blood glucose; TSH: thyroid stimulating hormone; FSH: follicle stimulating hormone; LH: luteinizing hormone

**Table 2:** GPR54 and Kiss1 gene polymorphisms in the study population.

Gene	Position	Allele	Location	dbSNP ID	A.A change	MAF in Samples
GPR54	917526	A→G	Exon 1	rs10407968	Gly →Gly	19.71%
	(GRCh38.p7)					
GPR54	918731	T→G	Intron 2	rs1250729403	-	11.83%
	(GRCh38.p7)					
GPR54	920170	G→T	Intron 4	rs350131	-	42.65%
	(GRCh38.p7)					
GPR54	918686	A→G	Intron 2	-	-	14.50%
	(GRCh38.p7)					
GPR54	918735	A→G	Intron 2	-	-	25.74%
	(GRCh38.p7)					
Kiss1	204196483	CTT→CT	5 prime UTR variant	rs5780218	-	21.76%
	(GRCh38.p7)					
Kiss1	204190659	C→G	Exon 3	rs4889	Pro→Arg	21.18%
	(GRCh38.p7)					

MAF: minor allele frequency.

**Table 3:** Genotypes distribution in cases and controls.

Gene	SNP	Genotype	Cases	Controls	p	OR (95% CI)
			(n=55)	(n=110)		
GPR54	rs10407968	A/G				
		AA	37 (67%)	75 (65%)		1^a^
		AG	18 (33%)	31 (27%)	0.649	0.850 (0.421 – 1.714)
		GG	00	09 (8%)	0.038	0.893 (0.829 – 0.962)
		AG + GG	18 (33%)	40 (35%	0.791	1.096 (0.555 – 2.167)
GPR54	rs1250729403	T/G				
		TT	40 (71%)	90 (78%)		1^a^
		TG	15 (27%)	25 (22%)	0.389	0.722 (0.344 – 1.517)
		GG	00	00		
GPR54	rs350131	G/T				
		GG	09 (16%)	24 (21%)		1^a^
		GT	26 (47%)	53 (46%)	0.557	0.764 (0.311 – 1.877)
		TT	20 (36%)	38 (33%)	0.478	0.713 (0.279 – 1.821)
		GT + TT	46 (84%)	91 (79%)	0.487	0.742 (0.319 – 1.726)
GPR54	Chr19: 918686	A/G				
	Gene:6345					
		AA	37 (67%)	84 (73%)		1^a^
		AG	18 (33%)	31 (27%)	0.394	0.738 (0.367 – 1.486)
		GG	00	00		
GPR54	Chr19: 918735	A/G				
	Gene:6394					
		AA	26 (47%)	56 (49%)		1^a^
		AG	28 (51%)	59 (51%)	0.947	0.978 (0.512 – 1.868)
		GG	00	00		
KISS1	rs5780218	CTT/CT				
		CTT/CTT	37 (67%)	68 (59%)		1^a^
		CTT/CT	14 (25%)	42 (36%)	0.184	1.632 (0.790 – 3.372)
		CT/CT	04 (7%)	05 (4%)	0.581	0.680 (0.172 – 2.688)
		CTT/CT+ CT/CT	18 (32%)	47 (40%)	0.307	1.421 (0.723 – 2.790)
KISS1	rs4889	C/G				
		CC	38 (69%)	67 (58%)		1^a^
		CG	16 (29%)	42 (36%)	0.264	1.489 (0.739 – 2.998)
		GG	01 (2%)	06 (5%)	0.239	3.403 (0.395 – 29.334)
		CG + GG	17 (31%)	48 (41%)	0.174	1.601 (0.810 – 3.166)

OR: odds ratio; CI: confidence intervals; ^a^  reference genotype.

**Table 4:** Association between SNPs of GPR54 gene and PCOS risk under multiple models of inheritance.

SNP	Model	Genotype	Cases	Controls	OR (95%CI)	p	AIC	BIC
rs10407968	Co-dominant	A/A	38 (69.1%)	73 (66.4%)	1.00			
		A/G	17 (30.9%)	28 (25.4%)	0.86 (0.42-1.76	0.021	208.3	217.6
		G/G	0 (0%)	9 (8.2%)	NA (0.00-NA)			

	Dominant	A/A	38 (69.1%)	73 (66.4%)	1.00	0.72	213.9	220.1
		A/G-G/G	17 (30.9%)	37 (33.6%)	1.13 (0.57-2.27)			

	Recessive	A/A-A/G	55 (100%)	101 (91.8%)	1.00	0.006	206.5	212.7
		G/G	0 (0%)	9 (8.2%)	NA (0.00-NA)			

	Overdominant	A/A-G/G	38 (69.1%)	82 (74.5%)	1.00	0.46	213.5	219.7
		A/G	17 (30.9%)	28 (25.4%)	0.76 (0.37-1.56)			

	Log-additive				1.39 (0.78-2.50)	0.25	212.7	219

rs1250729403		A/A	40 (72.7%)	81 (73.6%)	1.00	0.9	214	220.2
		A/G	15 (27.3%)	29 (26.4%)	0.95 (0.46-1.98)			

rs350131		T/T	40 (72.7%)	90 (81.8%)	1.00	0.18	212.3	218.5
		T/G	15 (27.3%)	20 (18.2%)	0.59 (0.28-1.27)			

Chr19: 918686		A/A	26 (47.3%)	52 (47.3%)	1.00	1	214	220.3
		A/G	29 (52.7%)	58 (52.7%)	1.00 (0.52-1.91)			

	Co-dominant	T/T	19 (34.5%)	41 (37.3%)	1.00	0.068	210.7	220
Chr19: 918735		G/T	30 (54.5%)	43 (39.1%)	0.66 (0.32-1.36)			
		G/G	6 (10.9%)	26 (23.6%)	2.01 (0.71-5.69			

	Dominant	T/T	19 (34.5%)	41 (37.3%)	1.00	0.73	213.9	220.1
		G/T-G/G	36 (65.5%)	69 (62.7%)	0.89 (0.45-1.75)			

	Recessive	T/T-G/T	49 (89.1%)	84 (76.4%)	1.00	*0.043*	209.9	216.2
		G/G	6 (10.9%)	26 (23.6%)	2.53 (0.97-6.57)			

	Overdominant	T/T-G/G	25 (45.5%)	67 (60.9%)	1.00	0.06	210.5	216.7
		G/T	30 (54.5%)	43 (39.1%)	0.53 (0.28-1.03)			

	Log-additive				1.21 (0.77-1.90)	0.4	213.4	219.6

AIC, Akaike information criteria; BIC, Bayesian information criteria; CI, confidence interval; OR: odds ratio.

**Table 5:** Association between SNPs of Kiss1 gene and PCOS risk under multiple models of inheritance.

SNP	Model	Genotype	Cases	Controls	OR (95%CI)	p	AIC	BIC
rs5780218	Co-dominant	CTT/CTT	35 (63.6%)	59 (53.6%)	1.00	0.44	214.4	223.7
		CTT/CT	17 (30.9%)	45 (40.9%)	1.57 (0.78-3.15)			
		CT/CT	3 (5.5%)	6 (5.5%)	1.19 (0.28-5.05)			

	Dominant	CTT/CTT	35 (63.6%)	59 (53.6%)	1.00	0.22	212.5	218.8
		CTT/CT-CT/CT	20 (36.4%)	51 (46.4%)	1.51 (0.78-2.94)			

	Recessive	CTT/CTT-CTT/CT	52 (94.5%)	104 (94.5%)	1.00	1	214	220.3
		CT/CT	3 (5.5%)	6 (5.5%)	1.00 (0.24-4.16)			

	Overdominant	CTT/CTT-CT/CT	38 (69.1%)	65 (59.1%)	1.00	0.21	212.5	218.7
		CTT/CT	17 (30.9%)	45 (40.9%)	1.55 (0.78-3.08)			

	Log-additive				1.33 (0.76-2.33)	0.31	213	219.2

	Co-dominant	C/C	36 (65.5%)	67 (60.9%)	1.00	0.61	215	224.4
rs4889		C/G	18 (32.7%)	38 (34.5%)	1.13 (0.57-2.27)			
		G/G	1 (1.8%)	5 (4.5%)	2.69 (0.30-23.88)			

	Dominant	C/C	36 (65.5%)	67 (60.9%)	1.00	0.57	213.7	219.9
		C/G-G/G	19 (34.5%)	43 (39.1%)	1.22 (0.62-2.39)			

	Recessive	C/C-C/G	54 (98.2%)	105 (95.5%)	1.00	0.35	213.2	219.4
		G/G	1 (1.8%)	5 (4.5%)	2.57 (0.29-22.57)			

	Over dominant	C/C-G/G	37 (67.3%)	72 (65.5%)	1.00	0.82	214	220.2
		C/G	18 (32.7%)	38 (34.5%)	1.08 (0.55-2.16)			

	Log-additive				1.27 (0.70-2.29)	0.43	213.4	219.6

AIC, Akaike information criteria; BIC, Bayesian information criteria; CI, confidence interval; OR: odds ratio.

**Table 6:** Haplotype frequencies of SNPs of GPR54 gene and association with PCOS.

rs10407968	rs1250729403	rs350131	Chr19:918686	Chr19:918735	Frequency	OR (95% CI)	p
A	A	T	A	T	0.311	1.00	- - -
G	A	T	A	G	0.1295	1.14 (0.45 - 2.89)	0.78
A	A	T	A	G	0.1244	1.59 (0.55 - 4.64)	0.39
A	A	T	G	T	0.1037	2.66 (0.61 - 11.60)	0.2
A	A	T	G	G	0.0735	1.32 (0.39 - 4.52)	0.66
A	G	T	A	T	0.0489	1.32 (0.26 - 6.56)	0.74
A	A	G	A	T	0.0403	3.02 (0.36 - 25.04)	0.31
A	A	G	A	G	0.0325	0.31 (0.05 - 1.92)	0.21
A	G	T	G	T	0.0323	0.33 (0.02 - 5.13)	0.43
A	G	T	A	G	0.0217		

**Table 7:** Haplotype frequencies of SNPs of Kiss1 gene and association with PCOS.

rs5780218	rs4889	Frequency	OR (95% CI)	p
CTT	C	0.6691	1.00	- - -
CT	C	0.1249	1.23 (0.60 - 2.54)	0.57
CT	G	0.1176	1.52 (0.64 - 3.61)	0.34
CTT	G	0.0885	1.11 (0.44 - 2.78)	0.83

## Data Availability

The data used to support the findings of this study are available from the corresponding author upon request.
